# Informativeness of Auditory Stimuli Does Not Affect EEG Signal Diversity

**DOI:** 10.3389/fpsyg.2018.01820

**Published:** 2018-09-26

**Authors:** Michał Bola, Paweł Orłowski, Karolina Baranowska, Michael Schartner, Artur Marchewka

**Affiliations:** ^1^Laboratory of Brain Imaging, Nencki Institute of Experimental Biology, Polish Academy of Sciences, Warsaw, Poland; ^2^Institute of Philosophy, University of Warsaw, Warsaw, Poland; ^3^Faculty of Electronics and Information Technology, Warsaw University of Technology, Warsaw, Poland; ^4^Faculty of Physics, Warsaw University of Technology, Warsaw, Poland; ^5^Département des Neurosciences Fondamentales, Université de Genève, Geneva, Switzerland

**Keywords:** consciousness, Lempel-Ziv complexity, signal diversity, speech processing, resting-state, EEG

## Abstract

Brain signal diversity constitutes a robust neuronal marker of the global states of consciousness. It has been demonstrated that, in comparison to the resting wakefulness, signal diversity is lower during unconscious states, and higher during psychedelic states. A plausible interpretation of these findings is that the neuronal diversity corresponds to the diversity of subjective conscious experiences. Therefore, in the present study we varied an information rate processed by the subjects and hypothesized that greater information rate will be related to richer and more differentiated phenomenology and, consequently, to greater signal diversity. To test this hypothesis speech recordings (excerpts from an audio-book) were presented to subjects at five different speeds (65, 83, 100, 117, and 135% of the original speed). By increasing or decreasing speed of the recordings we were able to, respectively, increase or decrease the presented information rate. We also included a backward (unintelligible) speech presentation and a resting-state condition (no auditory stimulation). We tested 19 healthy subjects and analyzed the recorded EEG signal (64 channels) in terms of Lempel-Ziv diversity (LZs). We report the following findings. First, our main hypothesis was not confirmed, as Bayes Factor indicates evidence for no effect when comparing LZs among five presentation speeds. Second, we found that LZs during the resting-state was greater than during processing of both meaningful and unintelligible speech. Third, an additional analysis uncovered a gradual decrease of diversity over the time-course of the experiment, which might reflect a decrease in vigilance. We thus speculate that higher signal diversity during the unconstrained resting-state might be due to a greater variety of experiences, involving spontaneous attention switching and mind wandering.

## Introduction

A key feature of our conscious experience is that each time point seems subjectively unique and different from other time-points. Therefore, a plausible hypothesis states that the diversity of subjective experiences is reflected by the diversity of brain activity patterns over time. A large repertoire of brain functional states is thus expected when a person is conscious, but not during loss of consciousness (Koch et al., 2016). Support for this hypothesis comes from studies in which transcranial magnetic stimulation (TMS) was used to activate specific brain regions, while electroencephalography (EEG) tracked the spread and diversity of neuronal activity (Massimini et al., 2005). A robust decrease of diversity of the TMS-evoked EEG response, as assessed by the Lempel-Ziv algorithm, has been demonstrated during NREM sleep (Casali et al., 2013; Pigorini et al., 2015), general anesthesia (Ferrarelli et al., 2010; Sarasso et al., 2015), and in patients with disorders of consciousness (Rosanova et al., 2012; Casarotto et al., 2016). Importantly, also Lempel-Ziv diversity (LZs) of the *spontaneous* EEG signal is decreased during sleep (Schartner et al., 2017b) and anesthesia (Schartner et al., 2015; fMRI: Hudetz et al., 2016). Further, evidence for reduced signal diversity during unconscious states has been also provided by studies investigating brain signals in terms of functional interactions (Bola et al., 2018), long-range temporal correlations (LRTCs) (Tagliazucchi et al., 2013; Krzemiński et al., 2017), or using dynamic systems theory measures (Solovey et al., 2015; Tajima et al., 2015). Interestingly, spontaneous electrophysiological signals exhibit higher LZs during psychedelic states induced by LSD, ketamine, or psilocybin (Schartner et al., 2017a; Wang et al., 2017). This demonstrates that not only decreases, but also increases from the “baseline” level of diversity are feasible, and suggests that changes in brain signal diversity might indeed reflect phenomenological diversity.

The studies conducted so far provide strong evidence that cortical signal diversity is associated with the global state of consciousness in human subjects. However, considering that transition between conscious and unconscious states affects nearly all aspects of neurophysiology and cognition, it is not known which specific mechanisms are associated with changes in signal diversity. Recent studies suggest that brain signal diversity is higher in response to meaningful stimuli, which evoke a range of different experiences in subjects perceiving them, and lower when processing stimuli without meaning, which do not result in differentiated experience (Boly et al., 2015; Mensen et al., 2017, 2018). Capitalizing on these observations we investigated a relation between signal diversity and the rate of information processed by a subject within a given time interval. Specifically, we hypothesized that greater information rate would result in richer and more differentiated phenomenology and, consequently, in greater neuronal diversity. This hypothesis is also in agreement with previously mentioned studies on global states of consciousness, as limited processing of external information during unconscious states (MacDonald et al., 2015) and altered information processing during psychedelic states (Carhart-Harris et al., 2014) might be the mechanisms behind, respectively, decreases and increases in brain signal diversity.

We therefore designed a study in which speech recordings (audio-books) were presented to subjects at different speeds (see Lerner et al., 2014). By increasing or decreasing the speed of recordings we were able to, respectively, increase or decrease the presented information rate. We also included backward presentations, in which sensory features of speech were preserved but the meaning (informativeness) was destroyed, and a resting-state without any task-related sensory stimulation. LZs of ongoing EEG was calculated using algorithms developed in previous studies (Schartner et al., 2017b) and compared among conditions with different information rate.

## Materials and Methods

### Subjects

We recruited 19 subjects (6 females; mean age 23,8 ± 2,3) who were native Polish speakers and had no history of neurological, neuropsychiatric, or hearing disorders. All subjects signed an informed consent document and received monetary reward (approximately 25 Euro). The procedure was approved by the Committee for Research Ethics of the Institute of Psychology of the Jagiellonian University (KE/08/012018).

### Procedure

We used fragments of a commercial audio-book red by a professional polish lector, which was based on a criminal novel “Röddin” (eng: “Voices”; pl: “Głos”) by Arnaldur Indri
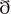
ason. When deciding on the auditory stimuli our aim was to choose an audio-book that first, was characterized by contemporary language and, second, had not been known to the participants before the experiment. All subjects confirmed that they had not read the novel or heard the audiobook before the experiment.

In the present study we aimed to manipulate the information rate that subjects processed by changing the speed of speech recordings. The audiobook was divided into 20 non-overlapping fragments with initial length of 140 s, so that after speeding up the length of the segment would be at least 90 s. Adobe Audition CC 2017 software was used to modify speed of the recordings. For every fragment we prepared the following versions: (1) original (100%) speed; (2) two slower versions (65 and 83% of the original speed); (3) two faster versions (117 and 135% of the original speed); and (4) an unintelligible (backward-played) version with the original 100% speed. In the experiment we also included a resting-state condition with no auditory stimulation. This resulted in seven experimental conditions to which we will refer as: *65*, *83*, *100*, *117*, and *135%*, *backward*, *resting-state*. The minimum and maximum speeds (*65* and *135%*, respectively) were chosen not to disturb comprehension of the material, in agreement with previous behavioral studies showing that speech can be understood even when played at twice or half of the original speed (Foulke, 1971; Wingfield, 1975).

The procedure was written in the Presentation Software 20.0 (Neurobehavioral Systems). During the experiment each condition was presented 4 times and the presentation time was 90 s for each condition (please note that we use the term “presentation” when referring to all conditions, including resting-state, even though nothing was presented during the resting-state). Thus for each condition we recorded 360 s of the EEG signal. The order of presentations and the assignment of specific audiobook fragments to conditions were randomized individually for each subject. Importantly, a given fragment of an audiobook was presented only once to each subject. During each condition a fixation cross was displayed in the center of the screen on a uniform gray background. Subjects were asked to fixate their gaze on the fixation cross throughout the experiment and listen attentively to audio recordings, or just fixate and sit still when there was no audio played (i.e., during resting-state). Short breaks were allowed between 90 s presentations and subjects were asked to resume the experiment by pressing a space bar whenever they felt ready. During breaks the fixation cross disappeared and there was no audio stimulation. Audio recordings were presented through the headphones and the volume was adjusted before the study began and kept constant for all subjects. The experiment lasted around 45 min and was conducted in a sound attenuated room.

### EEG Recording and Preprocessing

Electroencephalography was continuously recorded from 62 scalp sites and both ears (A1 and A2) using a 128-channels amplifier (Quick Amp, Brain Products) and the Brain Vision Recorder software (Brain Products). Ag-AgCl electrodes were mounted on an elastic cap (ActiCap) according to an extended 10–20 system. EEG signal was digitized at a 500 Hz sampling rate.

The following preprocessing steps were applied offline using custom-made Matlab scripts based on the EEGlab functions (Delorme and Makeig, 2004). Signals were filtered with a 1 Hz high-pass FIR filter (1,650 filter order), a 45–55 Hz notch FIR filter (826 filter order), and a 100 Hz low-pass FIR filter (66 filter order). Data were then down-sampled to 250 Hz, divided into 10 s long epochs (252 epochs per subject) and visually screened for non-stereotypical artifacts. Based on the visual inspection we discarded 50,3 ± 35,2 epochs and 3,9 ± 2,1 channels per subject. Next, signals were re-referenced to the average of all channels and decomposed into independent components, the number of which was equal to the number of retained channels. Time-courses and topographic maps of ICs were assessed to identify artefactual components and subtract them from the data (39,2 ± 11,8 components removed per subject). Missing channels were interpolated and, as the final step, the data were re-referenced to an average of A1 and A2 activity.

### Lempel-Ziv Analysis

**FIGURE 1:**
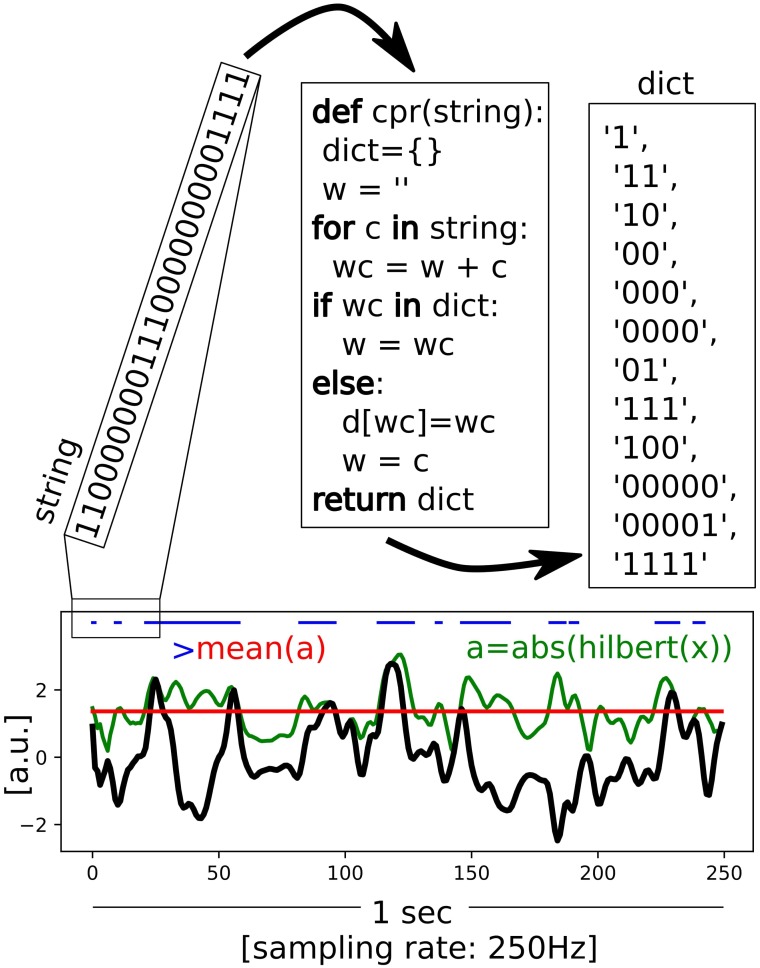
Scheme of the Lempel-Ziv diversity (*LZs*) calculation. The Hilbert transform is applied to the preprocessed EEG signal (in black) to estimate an envelope (green), which indicates amplitude of the signal at any given time-point. Based on the mean value (red) the envelope is binarized (blue). An application of the encoding step of the Lempel-Ziv algorithm is shown schematicaly for the first 25 points of the binarised string. The total number of unique patterns (“words”) is used as a way to quantify temporal diversity of the signal.

Single channels Lempel-Ziv diversity (*LZs*; **Figure 1**; Lempel and Ziv, 1976) was used to assess EEG signal diversity, using the implementation described in Schartner et al. (2017b). Briefly, the signal diversity of each EEG channel was assessed independently by demeaning (within 10 s long segment), dividing by standard deviation, and removing a linear trend. Hilbert transform was used to estimate the envelope of a signal (an absolute value of the analytic signal) and the mean of the envelope was used as a threshold to binarize the signal. The binarized signal was then segmented into blocks using the encoding step of the Lempel-Ziv compression algorithm. The number of blocks is the raw *LZs* score for the segment. Next the raw *LZs* value was normalized by the raw *LZs* value obtained from the same binary signal shuffled in time. Thus, the final (normalized) *LZs* score varies between 0 and 1 indicating, respectively, minimally and maximally diverse signals.

Another version of Lempel-Ziv complexity, denoted here by *LZc*, was used to capture signal diversity over both space and time simultaneously. In the computation of *LZc*, the 64 time series (one for each EEG channel) from a 10 s segment of data are binarized as described above for *LZs*, then concatenated observation-by-observation into one binary string such that the first 64 digits of that string are the observations of the 64 channels at time step 1, the next 64 are those at time step 2, etc. The diversity of this binary string is then assessed in the same way as described for *LZs* above.

### Statistical Analysis

The diversity measures, *LZc* and *LZs*, were compared among conditions in two statistical models. The first model tested the effect of information rate on diversity and thus included presentation speed as an independent variable with five levels. The second model tested the effect of processing meaningful information (*100%* condition) in comparison to *backward* and *resting-state*. Using Kolmogorov–Smirnov test (Matlab function *kstest*) we found that within all conditions the distribution of both measures is not Gaussian (2 × 7 tests conducted, all *p* < 0.05). Thus, the Friedman test (function *friedman*) was used as a non-parametric alternative to the repeated-measures ANOVA. When the result of a Friedman test was significant (*p* < 0.05) the non-parametric Wilcoxon signed-rank test (function *signrank*) was used to conduct *post hoc* comparisons between specific conditions. All statistical tests were done pair-wise with respect to the subjects, i.e., evaluating if the mean of the differences in scores between conditions is robustly different from zero. Bonferroni-Holm correction was applied to results of all the *post hoc* tests (Holm, 1979).

A third statistical model was used to test the possible effect of an increasing fatigue and loss of vigilance on diversity measures. To this end, for each subject all analyzed EEG segments were ordered based on the time of recording within the experiment, but irrespective of the condition, and divided into four quartiles. Thus, epochs recorded at the beginning of the experiment fall into Q1, while those recorded at the end into Q4. The effect of time the same statistical procedure as described in the first paragraph was used, with time being an independent variable with four levels (i.e., four quartiles).

The traditional null-hypothesis significance testing approach was complemented with Bayesian statistics in order to enable testing for the lack of differences between variables. The Bayes Factor (BF_10_) is defined as the ratio of the probability of observing the data given the alternative hypothesis is true, to the probability of observing the data given the null hypothesis is true. Thus BF_10_ provides a continuous measure of evidential support and is typically interpreted as follows (see Lee and Wagenmakers, 2013); BF_10_ < 0.1 indicates strong evidence in favor of the null hypothesis (i.e., lack of an effect); 0.1 < BF_10_ < 0.33 indicates moderate evidence in favor of the null hypothesis; 0.33 < BF_10_ < 3 indicates inconclusive data; 3 < BF_10_ < 10 indicates moderate evidence in favor of the alternative hypothesis (i.e., presence of an effect); and BF_10_> 10 indicates strong evidence in favor of the alternative hypothesis. Bayesian equivalents of a repeated-measures ANOVA and a paired-samples *t*-test with a medium prior scale (Cauchy scale: 0.707) were calculated using the JASP software (Wagenmakers et al., 2018).

When conducting the topographic analysis (**Figure 3**) statistical comparisons between a *100%* condition and a *resting-state* condition were conducted using cluster-based permutation test (Maris and Oostenveld, 2007). We used the implemantation of the test from the Mass Univariate ERP toolbox (Groppe et al., 2011; *clust_perm1* function).

## Results

In the present study we investigated diversity of EEG signals recorded during conditions with different information rates. Two indexes of diversity were analyzed: first, diversity of the spatio-temporal patterns estimated by applying the Lempel-Ziv algorithm to multi-channel EEG signals (termed *LZc* here); second, diversity of the temporal patterns estimated by applying the algorithm to single channel EEG signals (*LZs*, **Figure 1**).

**FIGURE 2:**
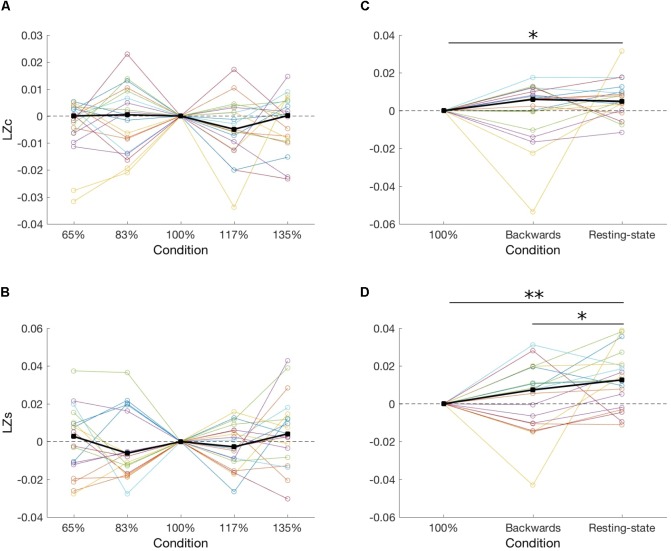
Two diversity measures, *LZc* and *LZs*, ploted across five presentation speeds of meaningful audio recordings **(A,B)** and for meaningful (100%), unintelligible (*backward*), and no-task (*resting-state*) conditions **(C,D)**. Each line represents one subject, and thick black lines indicate median values for each condition. In all panels results were normalized to the 100% condition by subtracting the 100% value from all conditions for each subject. ^∗^*p* < 0.05; ^∗∗^*p* < 0.01.

First, in order to test our hypothesis stating that neuronal diversity is greater in conditions with greater information rate, we compared the two diversity measures among five speeds of an audio presentation. However, we found that the speed of recordings had no significant effect, neither on *LZc* [*F*(4,14) = 1.18, *p* = 0.88; **Figure 2A**], nor on *LZs* averaged over all channels [*F*(4,14) = 3.49, *p* = 0.47; **Figure 2B**]. Importantly, Bayesian analysis indicates evidence in favor of the null hypothesis (BF_10_ = 0.14 and 0.24 for, respectively, LZc and LZs).

Second, we compared signal diversity during processing of meaningful auditory material (*100%*), meaningless auditory material (*backward*), and a condition of no auditory presentation (*resting-state*). We found a significant effect of a condition on *LZc* at the group level [*F*(2,16) = 6.73, *p* = 0.034; BF_10_ = 0.55; **Figure 2C**]. Further *post hoc* tests indicate that *LZc* during a *resting-state* was greater than during a *100%* condition [*Z*(18) = 2.45, *p* = 0.042; BF_10_ = 4.02]. Next, we also found a significant effect of condition on *LZs* [*F*(2,16) = 8.0, *p* = 0.018; BF_10_ = 3.92; **Figure 2D**]. Specifically, *LZs* during a *resting-state* was greater than during a *100%* condition [*Z*(18) = 3.0, *p* = 0.002; BF_10_ = 26.89] and a *backward* condition [*Z*(18) = 2.37, *p* = 0.035; BF_10_ = 1.10]. Of note, BF_10_ indicates evidence for no difference between meaningful (*100%*) and unintelligible (*backward*) speech (LZc: BF_10_ = 0.24; LZs: BF_10_ = 0.29), which is in disagreement with three recently published studies (Boly et al., 2015; Mensen et al., 2017, 2018).

**FIGURE 3:**
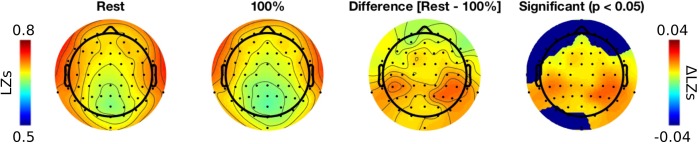
Topographic analysis of *LZs* during *resting-state* and 100*%* audio conditions.

Third, we conducted a topographic analysis of *LZs* by plotting topo maps for *resting-state* and *100%* conditions, and testing a difference between them using a cluster-based permutation test (Maris and Oostenveld, 2007; Groppe et al., 2011). Inspecting the topo-plots we noticed that temporal and parietal electrodes exhibit most robust differences between conditions. The cluster-based permutation test indicates a significant effect at nearly all electrodes (**Figure 3**). Additionally, we noticed that in both conditions temporal electrodes (T7, T8, TP9, TP10, FT7, FT8, TP7, TP8) were characterized by greatest *LZs* values. We suspect this can be attributed to the presence of miographic noise, which often contaminates signal at these electrodes (even after extensive preprocessing conducted in the present study) and might result in greater diversity of the signal patterns. Importantly, differences between conditions were strongest for parietal electrodes, and thus are unlikely to be explained by noise or other artifacts.

**FIGURE 4:**
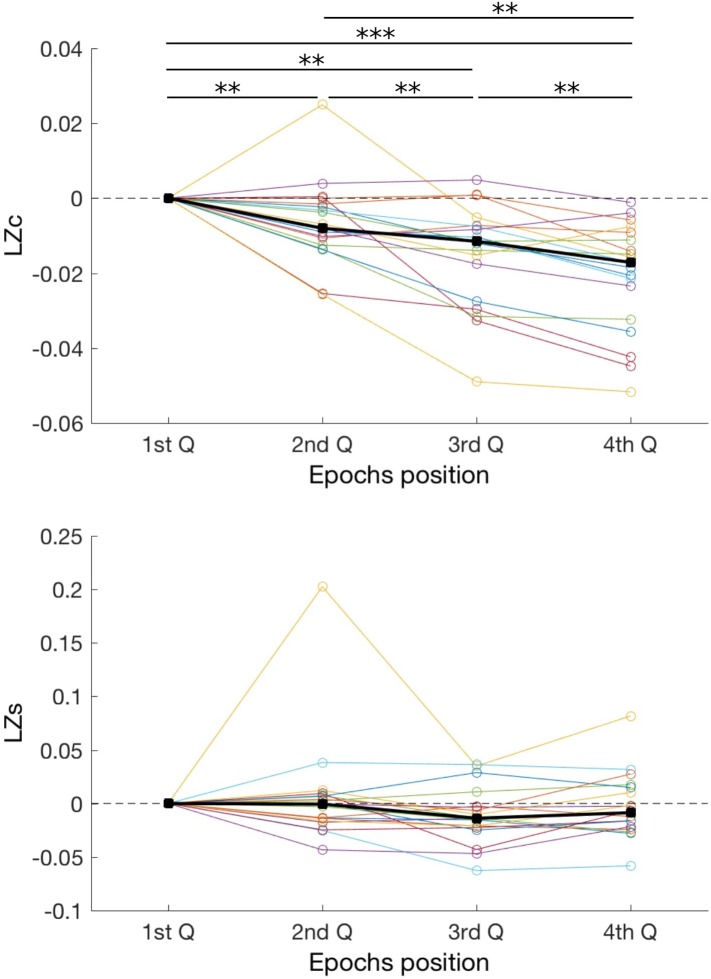
Changes in two diversity measures, *LZc* and *LZs*, over duration of the experiment. EEG segments were divided into four quartiles, based solely on the order within the experiment, and complexity of these segments was compared. As in **Figure 2**, each line represents one subject, and thick black lines indicate median values for each condition. For each subject results were normalized by subtracting the value obtained in the 1st quartile. ^∗∗^*p* < 0.01; ^∗∗∗^*p* < 0.001.

Finally, we conducted an additional analysis to investigate whether fatigue and loss of vigilance could have had an effect on diversity measures. To this end, for each subject we divided all analyzed EEG segments into quartiles, based solely on the order of recording during the experiment. Thus, Q1 are segments recorded at the beginning of the experiment, whereas Q4 recorded at the end (**Figure 4**). We hypothesized that a decrease of vigilance over time will be reflected by a decrease of signal diversity. Indeed, we found that time had strong effect on *LZc* [*F*(3,15) = 34, *p* < 0.001; BF_10_ > 100]. *Post hoc* comparisons show that *LZc* decreased over time, with comparisons between all quartiles being significant (all *p* < 0.01; all BF_10_ > 3). In case of *LZ*s, there was a significant, albeit weaker, effect of condition [*F*(3,15) = 9.6, *p* = 0.022; BF_10_ = 0.36] but none of the *post hoc* comparisons indicated significant difference (all *p* > 0.05; all 0.25 < BF_10_ < 1.5). Therefore, our analysis suggests that *LZc*, but less so *LZs*, is sensitive to the level of vigilance.

## Discussion

It has been comprehensively shown that brain signal diversity constitutes a robust neuronal marker of the global states of consciousness (Casali et al., 2013; review: Koch et al., 2016; Storm et al., 2017). In comparison to the resting wakefulness, neuronal diversity is lower during unconscious states, like NREM sleep (Pigorini et al., 2015; Andrillon et al., 2016; Schartner et al., 2017b), general anesthesia (Ferrarelli et al., 2010; Sarasso et al., 2015; Schartner et al., 2015), or in patients with disorders of consciousness (Rosanova et al., 2012; Casarotto et al., 2016). However, a key step in establishing explanatory correlates of consciousness is to find a mapping between neuronal processes and phenomenology (Seth, 2009). Indeed, recent finding of higher signal diversity during psychedelic experiences might be a first step toward this direction by suggesting that diversity measures might be sensitive to changes in the content of consciousness (Schartner et al., 2017a; Wang et al., 2017). Assuming that consciousness is a multidimensional phenomenon (Bayne et al., 2016; Fazekas and Overgaard, 2017; Jonkisz et al., 2017) one might then ask which of the proposed dimensions of experience are correlated with the signal diversity. In the present study we manipulated an information rate presented to the subjects and hypothesized that greater information rate will be related to richer and more differentiated phenomenology and, consequently, to greater EEG signal diversity. However, contrary to our hypothesis, we did not find any effect of the information rate on signal diversity. We also did not find differences when comparing processing of meaningful and unintelligible (backward played) speech. This demonstrates that the auditory information rate, or even complete lack of informativeness, does not affect EEG diversity. However, we did find that signal diversity during the resting-state condition (no auditory stimulation) was greater than during listening to both intelligible and unintelligible speech.

### No Information Rate Effect on Signal Diversity

In the present study we assumed that an increase in subjective differentiation of experience can result from one of the two phenomena – either from a decrease of the average duration of conscious percepts (perceptual moments) and/or from an increase of the qualitative differences across percepts (Schartner, 2017). Relating to the former, a key concept for our study is a temporal receptive field (TRF), defined as an interval within which brain areas temporally integrate information (review Hasson et al., 2015). TRF are not temporally fixed but can adjust to the dynamics of the environment (Ossmy et al., 2013). Crucially, Lerner et al. (2014) used similar speech recording manipulations (as used in our study) and demonstrated that TRFs extend when the information rate is lower, and contract when it is higher. Therefore, we reasoned that shorter/longer TRFs might result in shorter/longer conscious percepts (perceptual moments; Wittmann, 2015; White, 2017), and thus correlate with signal diversity. But this putative effect of the information rate on perceptual moments has yet to be directly demonstrated.

Concerning the second way to increase subjective differentiation, namely an increase of the qualitative differences across percepts, we reasoned that more information within a given TRF would result in richer and more informative experience. In our study we used 90 s presentations in all conditions, thus the speeded condition involved presentation of more perceptual scenes than the slowed condition. Nonetheless, greater richness of perception defined in this way did not result in greater EEG diversity. To explain lack of the effect one can refer to models assuming levels of perceptual processing (e.g., Hochstein and Ahissar, 2002; Kouider et al., 2010). According to these models, less time to process given perceptual scene might result in representing either fewer details, or representing perceptual rather than semantic features. In this scenario the incoming (presented) information rate varied, but the range of consciously perceived qualia could have been similar across conditions.

Evidence supporting a link between phenomenological and EEG signal diversity is provided by Canales-Johnson et al. (2017), who show that perceiving a bistable auditory stimulus as one percept results in higher integration (information sharing) and lower differentiation (diversity), whereas perceiving it as two percepts results in lower integration and higher differentiation. Further, Schartner et al. (2017a) found that greater MEG signal diversity, specifically single-channel Lempel-Ziv (*LZs*), was associated with greater subjective intensity of experience during a psychedelic state (as measured by a questionnaire; see also Tagliazucchi et al., 2014). The fact that subjects characterized their perception with statements such as: “I saw movements in things that were not actually moving,” “things looked strange,” or “I felt unusual bodily sensations” (Muthukumaraswamy et al., 2013) implies that the psychedelic phenomenology might be interpreted as having a wider variety of experiences in a given period of time. However, using the information rate manipulation we did not find its relation with signal diversity.

### No Effect of Meaningfulness of Stimuli on Signal Diversity

Two recent studies indicate that diversity of brain activity is greater when subjects perceive meaningful (and thus informative) visual stimuli than when they perceive stimuli matched regarding their sensory features but without any meaning (uninformative; Boly et al., 2015; Mensen et al., 2017). More specifically, Boly et al. (2015) showed that LZs of fMRI BOLD signals was lowest when subjects watched meaningless “TV noise,” intermediate for a movie cut into short randomized fragments, and highest for an original version of the movie. Of note, Boly et al. (2015) reported recording also resting-state data, but complexity of the resting-state condition is unfortunately not analyzed/reported in the paper. Further, Mensen et al. (2017) demonstrated that perceiving pictures that are meaningful (even when presenting various exemplars of the same category) results in greater differentiation of EEG event-related responses than perceiving meaningless pictures (even though they varied in their low-level sensory features). Therefore, our finding of lack of differences in neuronal diversity between meaningful and unintelligible (backward) audio stimuli, which is supported by Bayesian statistics, is at odds with results of these two studies. How can this be explained? First, there are methodological differences, as Boly et al. (2015) also analyzed an “ongoing” brain activity, but recorded with fMRI, whereas Mensen et al. (2017) used EEG, but recorded in an event-related design. Here we analyzed an “ongoing” EEG activity in the same way as in previous studies investigating diversity across global states of consciousness (Schartner et al., 2015, 2017a,b), but this type of analysis was not sensitive to differences between meaningful and meaningless auditory material. Second, in comparison to the visual “TV noise” condition used by Boly et al. (2015), the audio *backward* condition used here comprises more structure, which might partially explain why we did not find a decrease of diversity during a *backward* condition. This might also suggest that the basic sensory structure in the stimulus is sufficient to observe signal diversity comparable to meaningful stimuli. Future studies might additionally use an auditory white noise condition, which would be better matched to the visual “TV noise” condition. Finally, the majority of studies seeking neuronal correlates of consciousness, including Boly et al. (2015) and Mensen et al. (2017), was conducted in the visual modality. Taking into account that humans strongly rely on vision and large parts of the cortex are devoted to visual processing, it is feasible that features of the visual material have greater impact on global cortical signal diversity than features of the auditory material. This puts even stronger emphasis on the need to investigate other modalities and identify modality-specific and modality-independent mechanisms (Snyder et al., 2015).

### Higher Signal Diversity During Rest

The unexpected finding of our study is that LZs is greater during the resting-state than during listening to the meaningful or meaningless speech recordings. We argue that from the phenomenological perspective listening to the speech stream, even though involving constant changes in the content (i.e., in the presented perceptual scenes), might have been considered one “type” of experience. Conversely, more diverse “types” of experiences occur during the unconstrained resting-state – as attention spontaneously wanders between external stimuli from different modalities, internal bodily sensations, and spontaneously generated thoughts – which might result in the overall more differentiated experience. Taking this into account, we speculate that EEG diversity measures might correlate with phenomenological diversity but on a more coarse level than assumed by our initial hypothesis. Namely, that EEG signal diversity might reflect the diversity of “types” rather than content of experiences.

This interpretation remains speculative as the main caveat of our study is that subjective experience was not assessed. However, to our knowledge, there is no standardized tool allowing assessment of the subjective differentiation of experience over time that could have been used in the present study. In the future, experience sampling paradigms might be employed to test whether experiencing spontaneous thoughts is related to increases in signal diversity (Andrews-Hanna et al., 2018). Another avenue to link signal diversity with phenomenology is through questionnaires, which subjects’ can use to self-evaluate their experience during a resting-state, for example the Amsterdam Resting State Questionnaire (Diaz et al., 2013, 2014; Pipinis et al., 2017).

Apart from the phenomenological interpretation, ample studies reported that brain networks are activated differently during perceptual or cognitive tasks and the resting-state. Specifically during the resting-state the Default Mode Network (DMN) is activated, whereas task-positive networks are deactivated (Shulman et al., 1997; Greicius et al., 2003; Fox et al., 2005). Therefore, activity of these networks might contribute in different ways to neurophysiological diversity. Further, studies looking at functional connectivity networks from the graph theory perspective found that, in contrast to the resting-state, during task performance global integration of functional networks increases and local segregation decreases (Kinnison et al., 2012; Bola and Sabel, 2015; Finc et al., 2017; review Bola and Borchardt, 2016; Shine and Poldrack, 2017). Thus, greater integration among brain areas likely causes signals to become more interdependent and similar, which is in line with lower diversity during task performance. Finally, another line of studies investigated brain signals in terms of LRTCs and, in line with our results, observed stronger LRTCs during the resting-state and weaker during performance of a demanding task (He et al., 2010; He, 2011; Ciuciu et al., 2012). Taking into account that LRTCs also decrease during loss of consciousness (NREM sleep: Tagliazucchi et al., 2013; Anesthesia: Tagliazucchi et al., 2016; Krzemiński et al., 2017) they might capture similar features of neuronal dynamics as Lempel-Ziv, albeit on a longer time-scale.

### Decrease of Signal Diversity Over Time

In additional analyses we found a reliable decrease of signal diversity over time. Interestingly, this effect was found only for *LZc*, which quantifies diversity of the spatio-temporal patterns of brain activity, but not for *LZs*, which evaluates diversity solely over time. The drop of *LZc* was very robust, with all 19 subjects exhibiting lower *LZc* toward the end of the experiment (Q4) than at the beginning (Q1). We did not assess responsiveness of subjects continuously and thus we cannot exclude that some subjects fell asleep, but we think it is unlikely. We rather interpret this finding as related to decreasing arousal and vigilance while still being in the wakefulness state. If that is the case, this finding demonstrates that there might be reliable and meaningful changes in diversity even when changes in a behavioral state and neurophysiological activity patterns are relatively subtle (i.e., subtle in comparison to changes during loss of consciousness). Previously changes in EEG signal diversity across sleep stages have been described by Andrillon et al. (2016) and Schartner et al. (2017b).

Overall, in the present study we demonstrated that informativeness of the auditory stimuli did not affect the EEG signal diversity. Yet, we did find greater diversity during the resting-state than during attentive listening.

## Author Contributions

MB conceived and designed the study, analyzed and interpreted the data, and drafted and revised the article. PO prepared the experimental procedure, and collected, preprocessed, and analyzed the data. KB collected the data. MS developed the analysis methods, supervised the analysis, interpreted the data, and revised the manuscript. AM interpreted the data and revised the manuscript.

## Conflict of Interest Statement

The authors declare that the research was conducted in the absence of any commercial or financial relationships that could be construed as a potential conflict of interest.
